# Immunological characterization of stroke-heart syndrome and identification of inflammatory therapeutic targets

**DOI:** 10.3389/fimmu.2023.1227104

**Published:** 2023-10-30

**Authors:** Junyi Zheng, Yilin Ma, Xukun Guo, Jialing Wu

**Affiliations:** ^1^ Department of Cardiology, Tianjin Chest Hospital, Tianjin Institute of Cardiovascular Disease, Tianjin, China; ^2^ Chest Hospital, Tianjin University, Tianjin, China; ^3^ Clinical College of Neurology, Neurosurgery and Neurorehabilitation, Tianjin Medical University, Tianjin, China; ^4^ Department of Neurology, Department of Rehabilitation Medicine, Tianjin Neurosurgical Institute, Tianjin Huanhu Hospital, Tianjin Key Laboratory of Cerebral Vascular and Neurodegenerative Diseases, Tianjin, China

**Keywords:** stroke-heart syndrome, cardiac dysfunction, immune infiltration, inflammatory response, therapeutic targets

## Abstract

Acute cardiac dysfunction caused by stroke-heart syndrome (SHS) is the second leading cause of stroke-related death. The inflammatory response plays a significant role in the pathophysiological process of cardiac damage. However, the mechanisms underlying the brain–heart interaction are poorly understood. Therefore, we aimed to analysis the immunological characterization and identify inflammation therapeutic targets of SHS. We analyzed gene expression data of heart tissue 24 hours after induction of ischemia stoke by MCAO or sham surgery in a publicly available dataset (GSE102558) from Gene Expression Omnibus (GEO). Bioinformatics analysis revealed 138 differentially expressed genes (DEGs) in myocardium of MCAO-treated compared with sham-treated mice, among which, immune and inflammatory pathways were enriched. Analysis of the immune cells infiltration showed that the natural killer cell populations were significantly different between the two groups. We identified five DIREGs, *Aplnr*, *Ccrl2*, *Cdkn1a*, *Irak2*, and *Serpine1* and found that their expression correlated with specific populations of infiltrating immune cells in the cardiac tissue. RT–qPCR and Western blot methods confirmed significant changes in the expression levels of *Aplnr*, *Cdkn1a*, *Irak2*, and *Serpine1* after MCAO, which may serve as therapeutic targets to prevent cardiovascular complications after stroke.

## 1 Introduction

Many brain injuries such as stroke, traumatic brain injury (TBI), and various causes of intracranial hypertension can lead to arrhythmias, cardiac dysfunction, and heart failure (1). Ischemic stroke is a common clinical disease associated with high rates of disability and fatality; indeed, ischemic stroke is the main cause of death globally. Most deaths after stroke can be attributed to neurological damage, followed by cardiovascular complications (2). Acute ischemic stroke can induce cardiac dysfunction after the onset of neurological deficits even in patients without pre-existing cardiac co-morbidities, which Scheitz et al. has defined as stroke–heart syndrome (SHS) (3). The patients who suffer acute myocardial injury may have a higher short-term mortality rate than chronic myocardial injury (4). In fact, decreased cardiac function can be observed as early as 2 hours, obvious cardiac dysfunction and mortality was seen within the first 24h after stroke occurrence (5–7). Consequently, identifying targets for early changes and providing precise treatment is of great significance for saving heart function and reducing mortality.

Clinical and experimental evidence has demonstrated that inflammation plays a key role in heart damage caused by ischemic stroke. After stroke, local cerebral and systemic mediators mainly involving the autonomic system and inflammatory response can induce altered cardiomyocyte metabolism, changes in the composition of heart tissue-resident leukocyte populations, and microvascular changes (8). The inflammatory response cascade is essential for repairing and remodeling cardiac tissue, and non-selective suppression of inflammation can result in detrimental outcomes such as scarring, rupture, and exacerbation of adverse tissue remodeling (9). However, the precise molecular mechanisms that induce and regulate the inflammatory response after stroke, and how these pathways lead to myocardial dysfunction, remain unclear (10).

Although the link between stroke and cardiovascular complications is well recognized, there are inadequate data to establish effective treatment guidelines to manage cardiovascular outcomes after stroke (11). It remains challenging to distinguish between comorbid cardiovascular conditions and stroke-induced heart injury and provide individual treatment. To address the clinical need for protocols to quickly and accurately identify cerebral cardiac syndrome, here, we use experimental stroke models and bioinformatics methods to delineate the pathological mechanisms that drive SHS to identify the most relevant therapeutic targets.

## 2 Materials and methods

### 2.1 Animal model

To generate a mouse model of transient middle cerebral artery occlusion (MCAO), we induced focal cerebral ischemic stroke in 8-week-old male C57BL/6 mice (20–25 g) as described previously (12). In brief, the mice were anesthetized using sevoflurane, then an incision was made to allow separation of the cervical skin from the external carotid artery. Next, a nylon monofilament was inserted into the left internal carotid artery through the origin of the middle cerebral artery, where it remained for 1 h to induce cerebral ischemia. After the ischemic episode, mice were reperfused for 24 h. A decrease in cerebral blood flow >70% was considered a successfully induced MCAO model, and this was monitored using a laser Doppler flow meter (Perimed, Switzerland) and throughout cerebral reperfusion. The preparation for the sham surgery was only separated external carotid artery but no suture was placed. The number of the mice in the sham control group is 10, and in the SHS group is 20 in 24h and 10 in other time point groups, respectively, which are the ones that survived 24 h after transient MCAO.

### 2.2 2,3,5-Triphenyl-tetrazolium chloride staining

TTC (Merck, Darmstadt, Germany) staining was used to observe cerebral infract volume in MCAO mice. After 24 hours of reperfusion, mice were euthanized with a narcotic overdose and the brain was completely stripped. After freezing in at −20°C for 20 min, brain tissue was cut into 2-mm-thick slices. The tissue slices were then incubated in a 2% TTC solution for 15-30 min at 37°C to identify the cerebral infract.

### 2.3 Measurements of neurological deficits

Neurological deficits in MCAO mice were assessed using the modified neurological severity score (mNSS) test, as described previously (12). The highest score for the mNSS test is 18, and higher scores indicate more severe neurological deficit.

### 2.4 Serum troponin I mearsument

Serum cTnI in peripheral blood samples was measured using a mouse TNNI3/cTn-I (Troponin I Type 3, Cardiac) ELISA kit (Elabscience, Wuhan, China). According to the manufacturer’s protocol, after 100 μL each of blank, standard and sample were pipetted into individual wells and incubated at 37°C for 90 min, 100 μL of biotinylated detection Ab working solution was added to each well and incubated at 37°C for 1 h. After 3 washes, 100 μL horseradish peroxidase (HRP) Conjugate Working Solution was added to each well followed by another incubation at 37°C for 30 min. Then samples were washed 5 times and 90 μL of Substrate Reagent was added to each well. After a 15-minincubation at 37°C, 50 μL of Stop Solution was added to each well and the optical density (OD value) of each well was measured using a microplate reader (Molecular Devices, USA) set to 450 nm.

### 2.5 Immunofluorescence staining

After the mice were euthanized by excessive anesthesia, they were infused with 4% paraformaldehyde in the heart, then the hearts were collected and fixed in 4% paraformaldehyde. Subsequently, the hearts were dehydrated, paraffin embedded, cut into 3-mm-thick slices, and baked at 72°C for 1 h. Heart tissue slices were placed at room temperature for 10 minutes before dewaxing with water, and antigens were retrieved using EDTA (pH9.0) in a high-pressure chamber. Next, the tissue endogenous peroxidase was blocked with 3% hydrogen peroxide (ZSGB-Bio, Beijing, China) for 10 min at room temperature, followed by incubation with anti-CD45 antibody (Cell Signaling Technology (CST), MA, USA, 1:50 dilution) at 4°C overnight in the dark. The tissue was then incubated with goat anti-mouse Alexa Fluor 594 (Thermo Fisher, MA, USA, 1:500 dilution) at room temperature for 1 h and nuclei were stained with 4’,6-diamidino-2-phenylindole (DAPI) (Solarbio, Beijing, China). Tissue was mounted on slides with a coverslip and photographed using a laser-scanning confocal microscope (FluoView 1200, Olympus, Japan).

### 2.6 Data source and acquisition

To analyze the pathophysiological process of cardiac damage induced by ischemic stroke, we downloaded an expression profiling dataset (GSE102558) from Gene Expression Omnibus (GEO) database (https://www.ncbi.nlm.nih.gov/geo/), which was contributed by R. Veltkamp and based on the GPL20710 platform of [MoGene-2_0-st] Affymetrix Mouse Gene 2.0 ST Array (mogene20st_Mm_ENTREZG_19) and analyzed the expression data of heart tissue 24 hours after MCAO or sham surgery.

### 2.7 Identification of differentially expressed genes

DEGs were identified using the GEO2R (http://www.ncbi.nlm.nih.gov/geo/geo2r) online web tool provided by the GEO database. The parameters |log_2_fold change|≥1 and adjusted *P* value <0.05 were used as cut-offs for defining DEGs. A heatmap of the DEGs was constructed using the R package, “ComplexHeatmap”.

### 2.8 Functional enrichment analysis of DEGs

The Metascape (http://metascape.org/gp/index.html) online web tool was used to conduct Gene Ontology (GO) enrichment analysis, which includes molecular function (MF), biological process (BP), and cellular component (CC), as well as Kyoto Encyclopedia of Genes and Genomes (KEGG) pathway enrichment analysis (13, 14). The data were analyzed using the R packages “clusterProfiler” and “GOplot” and visualized by the R package “ggplot2”. *P*<0.05 and false discovery rate (FDR) <0.25 were set as the cut-off values.

### 2.9 Analysis of immune cells infiltration

To evaluate infiltrated immune cells propotions in cardiac tissue, the online bioinformatics algorithms CIBERSORTx (https://cibersortx.stanford.edu/index.php) (15) and mice leukocyte gene signature matrix (16) were used. Correlation analysis and visualization of immune cells were conducted using the R package, “ggplot2”.

### 2.10 Identification of differentially expressed inflammation-related genes

Inflammatory-related genes (IRGs), named “HALLMARK_INFLAMMATORY_RESPONSE” were obtained from the Molecular Signatures Database (MSigDB) (http://www.gsea-msigdb.org/) (17). The online tool Venn (http://DrawVennDiagram(ugent.be)) was used to identify differentially expressed IRGs (DIREGs) by identifying genes in the intersection of the Venn diagram. Correlations between DIREGs and visualization were analyzed using the R packages “igraph” and “ggraph”.

### 2.11 Real-time qPCR

TRIzol Reagent (Thermo Fisher, MA, USA) and reverse transcriptase (TransGen, Beijing, China) were used to extract total RNA from mice hearts and reverse-transcribe RNA into cDNA, respectively. A SYBR Green qPCR kit (TransGen, Beijing, China) was used to amplify *Aplnr*, *Ccrl2*, *Cdkn1a*, *Irak2*, *Serpine1* and *Gapdh* by RT-qPCR. The PCR system include 0.5 μL of cDNA, 0.1 μL each of upstream and downstream primers (100 μM), 7.5 μL of 2×SYBR Green qPCR reagent, and 6.8 μL of ddH_2_O. Three-step PCR program was carried out as follows: preheating program is at 94°C for 3 min, amplification program include forty cycles of denaturation at 95°C for 15 s and annealing at 62°C, and elongation program is at 75°C for 3 min. The relative expression level of each gene was calculated using the 2^-ΔΔCT^ method. The primers used were listed in **Table 1**.

**Table 1:** The primer sequences used for RT-qPCR.

Primer	Sequence (5′–3′)
Aplnr forward	TCGTGGTGCTTGTAGTGACC
Aplnr reverse	ATGCAGGTGCAGTACGGAAA
Ccrl2 forward	GACCAGCGCAGTTTCACTTT
Ccrl2 reverse	TCCATCGGAGGCTGTCCTTG
Cdkn1a forward	CAGACCAGCCTGACAGATTTCT
Cdkn1a reverse	GAGGGCTAAGGCCGAAGATG
Irak2 forward	GCTCAGGGCAACTCAGACAT
Irak2 reverse	CAGCAAGACGTTGGCACTCT
Serpine1 forward	GCACAACCCGACAGAGACA
Serpine1 reverse	ATGAAGGCGTCTCTTCCCAC
Gapdh forward	TTCACCACCATGGAGAAGGC
Gapdh reverse	GGCATGGACTGTGGTCATGA

### 2.12 Western blot

Samples used for Western Blot analysis were prepared following methods in our previous study (12). Briefly, after grinding, a portion of the heart tissue powder was incubated in RIPA buffer with a combination of phosphatase and protease inhibitors to lyse cells, and abicinchoninic acid (BCA) kit (Solarbio, Beijing, China) was used to quantify protein concentration. Then, an equal volume of each sample was denatured for 5 min at 95°C in loading buffer, and separated by sodium dodecyl sulfate-polyacrylamide gel electrophoresis (SDS-PAGE). Gels were transferred to a polyvinylidene fluoride membrane (PVDF), which were then blocked for 2 h in 5% bovine serum albumin (BSA) and incubated overnight at 4°C with the following primary antibodies: anti-IRAK2 (Cell Signaling Technology (CST), MA, USA, 1:1000 dilution), anti-APLNR, anti-CDKN1A, anti-SERPINE1 or anti-GAPDH (Proteintech, Rosemont, USA, 1:500, 1:1000, 1:10000,1:10000 dilution respevtively). Membranes were then washed three times with phosphate-buffered saline containing Tween 20 (PBST), and incubated for an hour with horseradish peroxidase (HRP) conjugated secondary antibody (Proteintech, Rosemont, USA, 1:10000 dilution). Immunoblots were visualized using a gel imaging system (Fusion FX7, ViIbertLourmat, France).

### 2.13 Statistical analyses

All data were processed using SPSS 22.0 statistical software. Comparisons between two groups were performed using an independent samples t-test, and correlation analyses were performed using Pearson’s method. One-way analysis of variance (ANOVA) was used for multiple-group comparisons, and the least significant difference *post-hoc* test was used for intergroup comparisons. *P* values < 0.05 were considered statistically significant.

## 3 Results

### 3.1 MCAO-induced cardiac dysfunction

**Figure 1:**
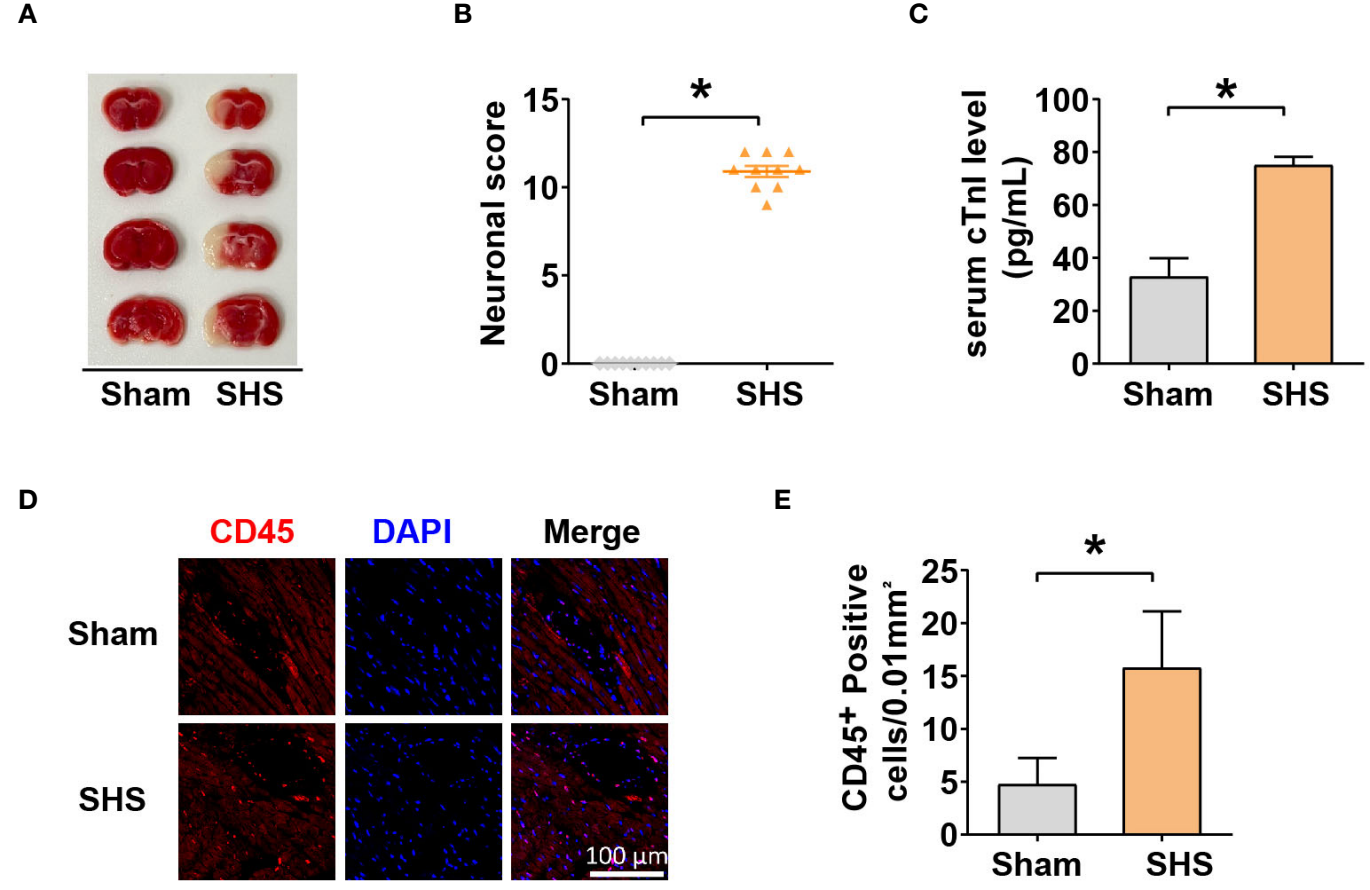
Stroke induced heart injury. **(A)** The cerebral infarct volume was determined by TTC staining. **(B)** mNSS scores of mice measured 24 h after MCAO. **(C)** Serum cTnI levels determined by ELISA. **(D–E)** Leukocyte infiltration into myocardium **(D)** and quantification **(E)** measured by IF with anti-CD45 antibody. *P<0.05.

To investigate molecular changes leading to SHS, MCAO was induced in mice for 1 h followed by reperfusion for 24 h. After the reperfusion period, we determined infarct volumes and neurological deficit scores. MCAO-treated mice had a significantly higher cerebral infarct volume and neurological deficit scores compared with sham-treated mice (**Figures 1A, B**). To examine whether MCAO induced cardiac damage, we next examined the level of serum cTnI, a marker of acute myocardial injury, which showed that MCAO-treated mice had significantly higher serum cTnI compared with sham-treated mice (**Figure 1C**). Furthermore, IF analysis revealed significantly increased leukocyte infiltration in the myocardial tissue of MCAO-treated mice compared with sham-treated mice (**Figures 1D, E**). Collectively, these data indicate that MCAO was successfully induced in mice and that MCAO-treated mice suffered acute cardiac dysfunction and inflammation.

### 3.2 Identification of DEGs in SHS

**Figure 2:**
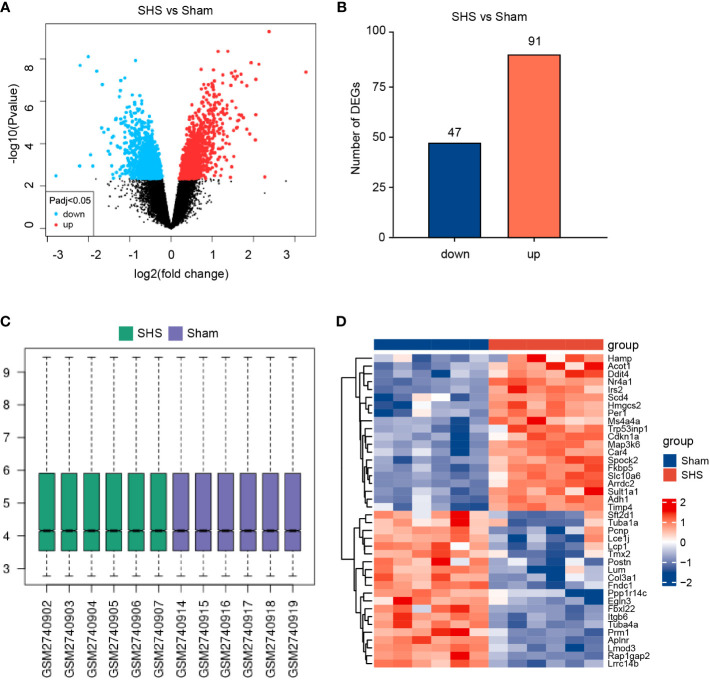
Identification of DEGs in the myocardium of SHS- and sham-treated mice in the GEO GSE102558 dataset. **(A)** Volcano plot of gene expression levels in SHS- and sham-treated mice. **(B)** A total of 138 DEGs were identified, including 91 upregulated genes and 47 downregulated genes in SHS- versus sham-treated mice. **(C)** The box diagram of data in the SHS and sham datasets. **(D)** Representative heatmap of the top 20 genes most upregulated and most downregulated DEGs.

To identify pathways that may contribute to SHS, we next evaluated gene expression profiles from a publicly available database of RNA expression profiling data of myocardial tissue from MCAO- and sham-treated mice after 24 h of perfusion. Applying the standard criteria of |log_2_fold change| ≥1 and adjusted *P* value < 0.05, we identified 138 DEGs, including 91 upregulated genes and 47 downregulated genes in MCAO-treated mice versus sham-treated mice (**Figures 2A, B**). The box diagram indicates that the data has been standardized and is suitable for analysis (**Figure 2C**). The top 40 DEGs are shown in **Figure 2D**.

### 3.3 Functional enrichment analysis

**Figure 3:**
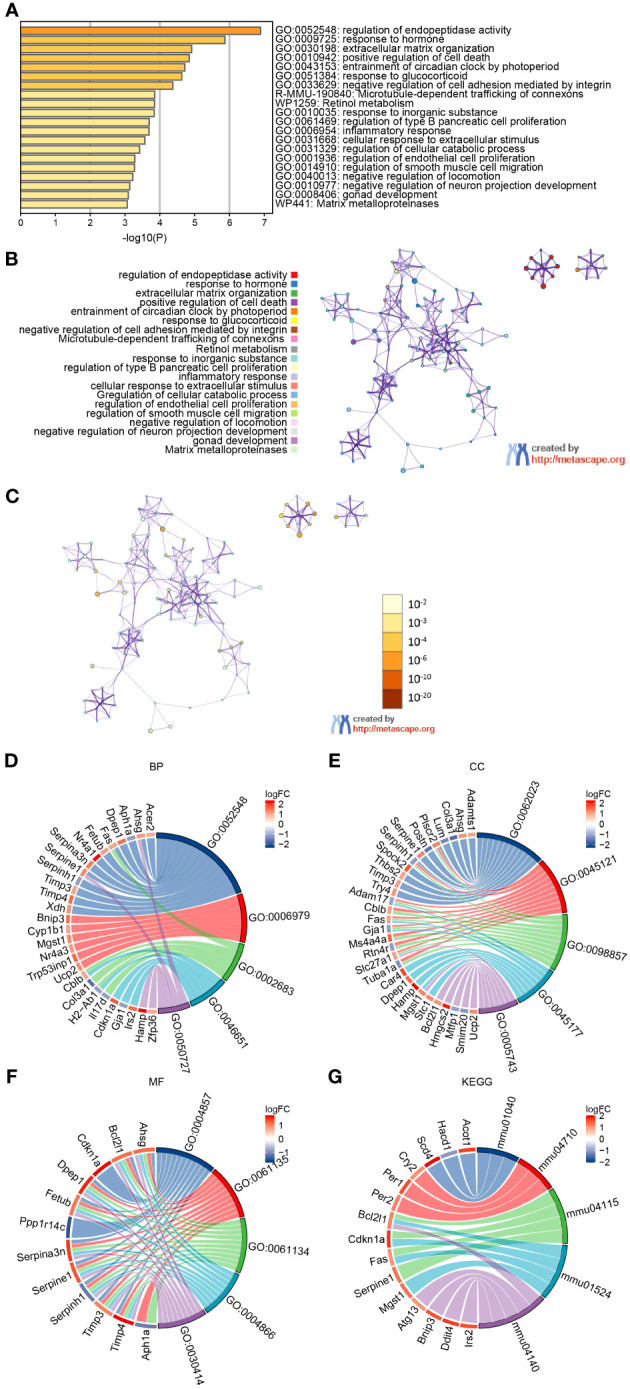
GO and KEGG analysis of DEGs in myocardium of MCAO- and sham-treated mice. **(A, B)** Significantly enriched pathways determined by GO and KEGG analysis using MetaScape. **(C)** The enriched ontology clusters colored by p-value. **(D–G)** Chordal graph of BPs **(D)**, CCs **(E)**, and MFs **(F)** from GO analysis. **(G)** Chordal graph of enriched pathways determined by KEGG analysis.

To understand the biological relevance of the DEGs for the development of SHS, we first analyzed the DEGs using the Metascape online tool (http://metascape.org). This showed that the DEGs were predominantly enriched in the regulation of endopeptidase activity, response to hormone, extracellular matrix organization, positive regulation of cell death, and entrainment of circadian clock by photoperiod (**Figures 3A, B**). The enriched ontology clusters colored by p-value is shown in **Figure 3C**. We then performed GO analysis on the DEGs and visualized the results using chord diagrams, which not only display the item, but also display the molecules contained within the item. In agreement with the MetaScape analysis, the BP changes of DEGs were significantly enriched in regulation of peptidase activity (GO:0052548), which includes the genes *Fas*, *Nr4a1*, *Serpina3n*, *Serpine1*, and others (**Figure 3D**). The response to oxidative stress (GO:0006979), negative regulation of immune system process (GO:0002683), lymphocyte proliferation (GO:0046651), and regulation of inflammatory response (GO:0050727) were also significantly enriched. The CC changes of DEGs were most enriched in collagen-containing extracellular matrix, membrane raft, membrane microdomain, apical part of cell, and mitochondrial inner membrane (**Figure 3E**). The MFs that were most enriched among the DEGs were enzyme inhibitor activity, endopeptidase regulator activity, peptidase regulator activity, endopeptidase inhibitor activity, and peptidase inhibitor activity (**Figure 3F**). Finally, we analyzed the DEGs using KEGG, which showed that the most enriched pathways were autophagy–animal (mmu04140), p53 signaling pathway (mmu04115), platinum drug resistance (mmu01524), biosynthesis of unsaturated fatty acids (mmu01040), and circadian rhythm (mmu04710) (**Figure 3G**). The terms of each category were listed in **Table 2**.

**Table 2:** GO and KEGG pathway enrichment analysis of DEGs.

Term	Description	P value	FDR
BP			
GO:0052548	regulation of endopeptidase activity	1.154E-08	2.224E-05
GO:0006979	response to oxidative stress	1.157E-02	6.214E-02
GO:0002683	negative regulation of immune system process	5.563E-02	1.380E-01
GO:0046651	lymphocyte proliferation	1.927E-02	8.336E-02
GO:0050727	regulation of inflammatory response	7.828E-02	1.497E-01
CC			
GO:0062023	collagen-containing extracellular matrix	2.885E-07	5.041E-05
GO:0045121	membrane raft	1.355E-05	8.069E-04
GO:0098857	membrane microdomain	1.385E-05	8.069E-04
GO:0045177	apical part of cell	1.807E-03	3.508E-02
GO:0005743	mitochondrial inner membrane	5.248E-03	6.113E-02
MF			
GO:0004857	enzyme inhibitor activity	4.248E-06	2.593E-04
GO:0061135	endopeptidase regulator activity	1.709E-07	5.218E-05
GO:0061134	peptidase regulator activity	6.010E-07	9.173E-05
GO:0004866	endopeptidase inhibitor activity	9.583E-07	9.751E-05
GO:0030414	peptidase inhibitor activity	1.296E-06	9.887E-05
KEGG			
mmu04140	Autophagy - animal	5.150E-03	1.897E-01
mmu04115	p53 signaling pathway	2.443E-03	1.500E-01
mmu01524	Platinum drug resistance	3.582E-03	1.650E-01
mmu01040	Biosynthesis of unsaturated fatty acids	2.363E-03	1.500E-01
mmu04710	Circadian rhythm	2.363E-03	1.500E-01

### 3.4 Immune cell infiltration in cardiac tissue

**Figure 4:**
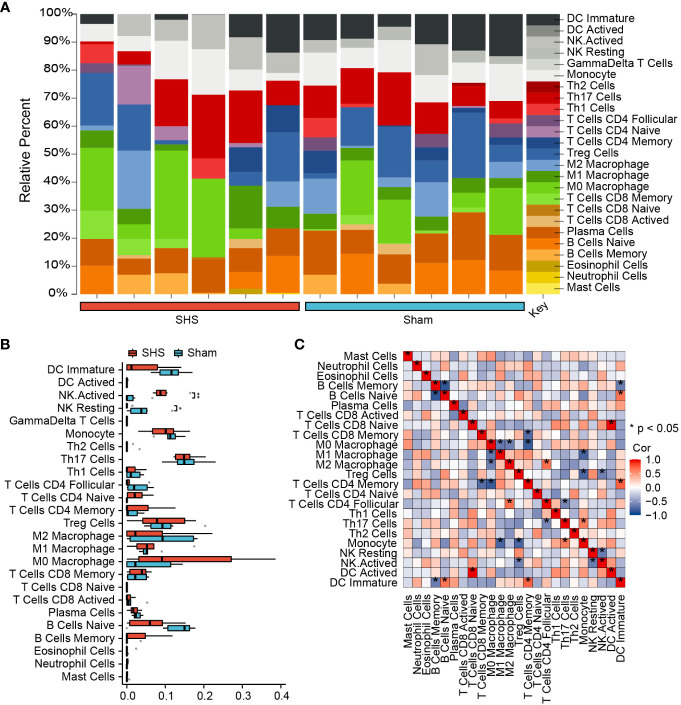
Analysis of immune cell infiltration in cardiac tissue of SHS- and sham-treated mice. **(A)** The fraction of 25 subsets of immune cells in SHS and sham samples. **(B)** The box graph shows the difference in populations of infiltrating immune cells between SHS and sham samples. **(C)** Correlation analysis of 25 types of immune cells. *P<0.05, **P<0.01.

Based on our experimental findings and the gene expression analysis, we next evaluated the immune cell infiltrate in cardiac tissues of MCAO- and sham-treated mice. We found that the proportion of resting natural killer (NK) cells was significantly lower in MCAO-treated compared with sham-treated mice (*P*=0.0263), whereas the proportion of activated NK cells was significantly higher compared with the sham-treated group (*P*=0.0043). Also, the proportion of B memory cells, M0 macrophages, and CD4^+^ naive T cells were significantly increased and the proportion of naïve B cells, CD4^+^ follicular T cells, and immature dendritic cells (DCs) were significantly decreased in MCAO-treated compared with sham-treated mice (**Figures 4A, B**). Correlation analysis of 25 types of immune cells determined that the strongest positive correlation was between activated DC cells and CD8^+^ naive T cells (*r*=1, *P*=0) and that the strongest negative correlation was between memory B cells and naive B cells (*r*=−0.85, *P*<0.001) (**Figure 4C**).

### 3.5 Identification of differentially expressed inflammation-related genes

**Figure 5:**
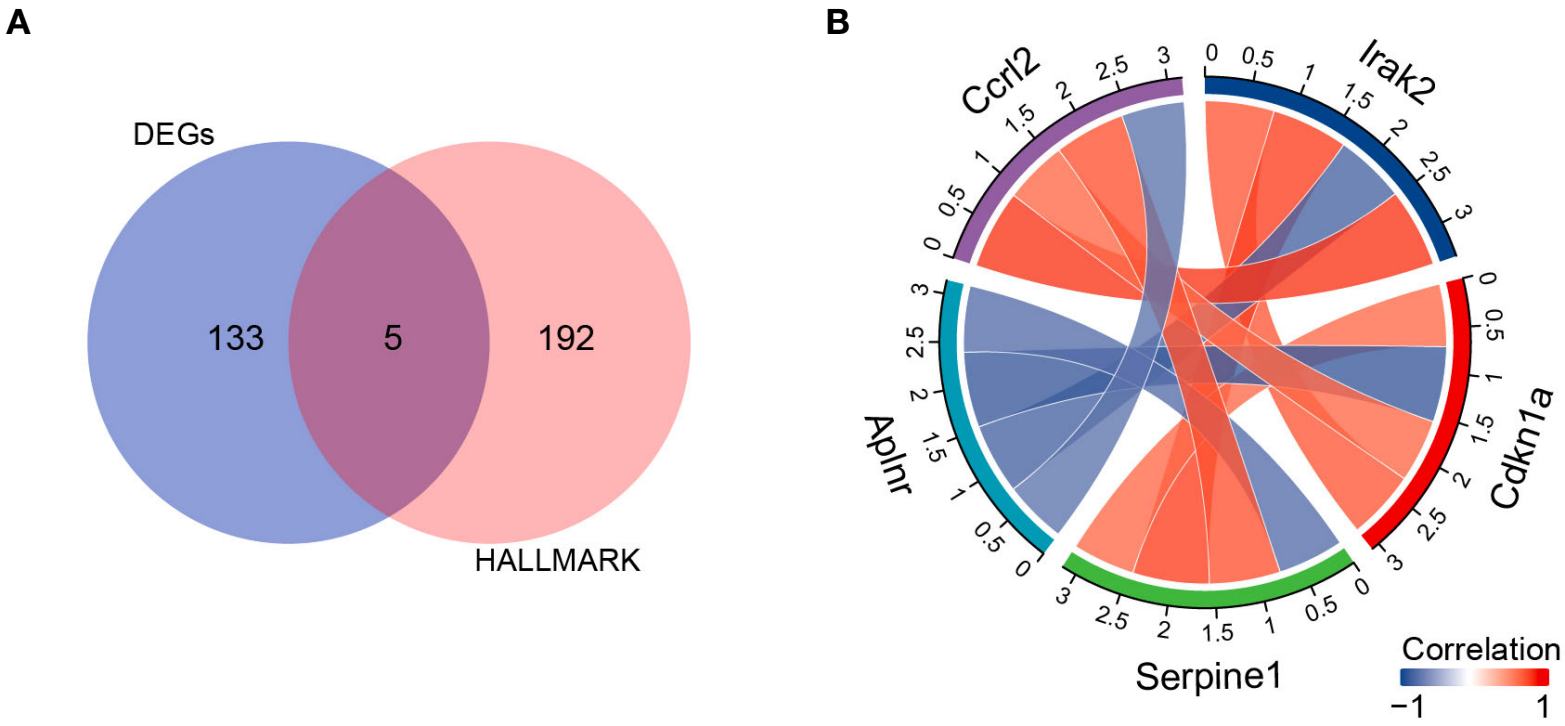
DIREGs in MCAO versus sham mouse myocardium. **(A)** Venn diagram showing intersection between138 DEGs and the HALLMARK_INFLAMMATORY_RESPONSEgenes to identify DIREGs. **(B)** Chord diagram of correlation analysis of DIREGs.

Next, we evaluated which inflammatory response genes may contribute to SHS. We identified 5 IRGs among our DEGs (DEIRGs): *Aplnr*, *Ccrl2*, *Irak2*, *Cdkn1a*, and *Serpine1* (**Table 3**). All of the DEIRGs except *Aplnr* were significantly upregulated in MCAO myocardium compared with sham myocardium. Correlation analysis showed that *Ccrl2* and *Irak2* were highly positively correlated (*r*=0.902), whereas *Aplnr* and *Cdkn1a* were highly negatively correlated (*r*=−0.860) (**Figure 5**).

**Table 3:** The 5 different inflammation-related expression genes.

Gene Symbol	adj.P.Val	P.Value	logFC	Changes
Aplnr	0.0000492	7.88E-09	-2.005466	Down
Ccrl2	0.0000832	4.66E-08	1.4669122	Up
Irak2	0.0012626	6.52E-06	1.0509508	Up
Cdkn1a	0.0012299	6.01E-06	1.7556017	Up
Serpine1	0.0056919	9.60E-05	1.3327433	Up

### 3.6 Correlation between DEIRGs and infiltrating immune cells

**Figure 6:**
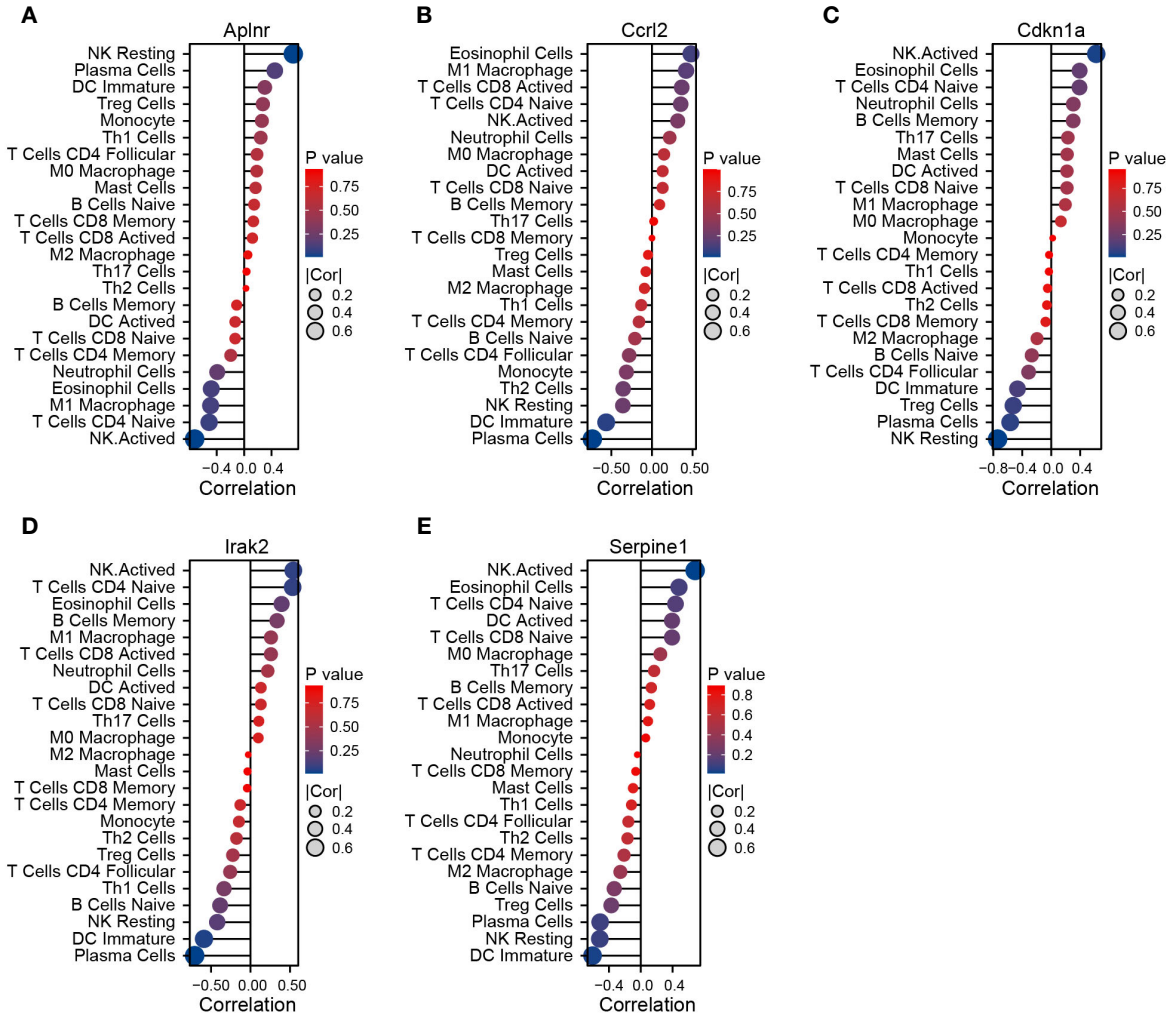
Correlation between DEIRGs and immune cells infiltrating the myocardium of MCAO-treated mice. **(A–E)** Correlation between expression of *Aplnr* **(A)**, *Ccrl2* **(B)**, *Cdkn1a* **(C)**, *Irak2* **(D)**, and *Serpine1* **(E)** and immune cell populations.

We used Pearson’s rank correlation analysis to explore the association between DEIRGs and the immune infiltrate in cardiac tissue of MCAO-treated mice. *Aplnr* expression was positively correlated with the proportion of infiltrating resting NK cells and negatively correlated with the proportion of infiltrating activated NK cells (**Figure 6A**). *Ccrl2* expression was positively correlated with the proportion of infiltrating eosinophil cells and negatively correlated with the proportion of infiltrating plasma cells (**Figure 6B**). *Cdkn1a*, *Irak2*, and *Serpine1* expression were positively correlated with the proportion of infiltrating activated NK cells and negatively correlated with the proportion of infiltrating resting NK, plasma cells and immature DC respectively (**Figures 6C–E**).

### 3.7 Validation of target genes

To validate the DEIRGs identified and further characterize their expression patterns during cardiac dysfunction, we performed RT-qPCR-based analysis of *Aplnr*, *Ccrl2*, *Cdkn1a*, *Irak2*, and *Serpine1* transcription levels at 1, 3, 6, 12 and 24 h after MCAO. The results showed that these five genes exhibited significant changes in expression in heart tissue following MCAO. It begins within 1 h after MCAO, continuously changing over the 24 h observation period.

**Figure 7:**
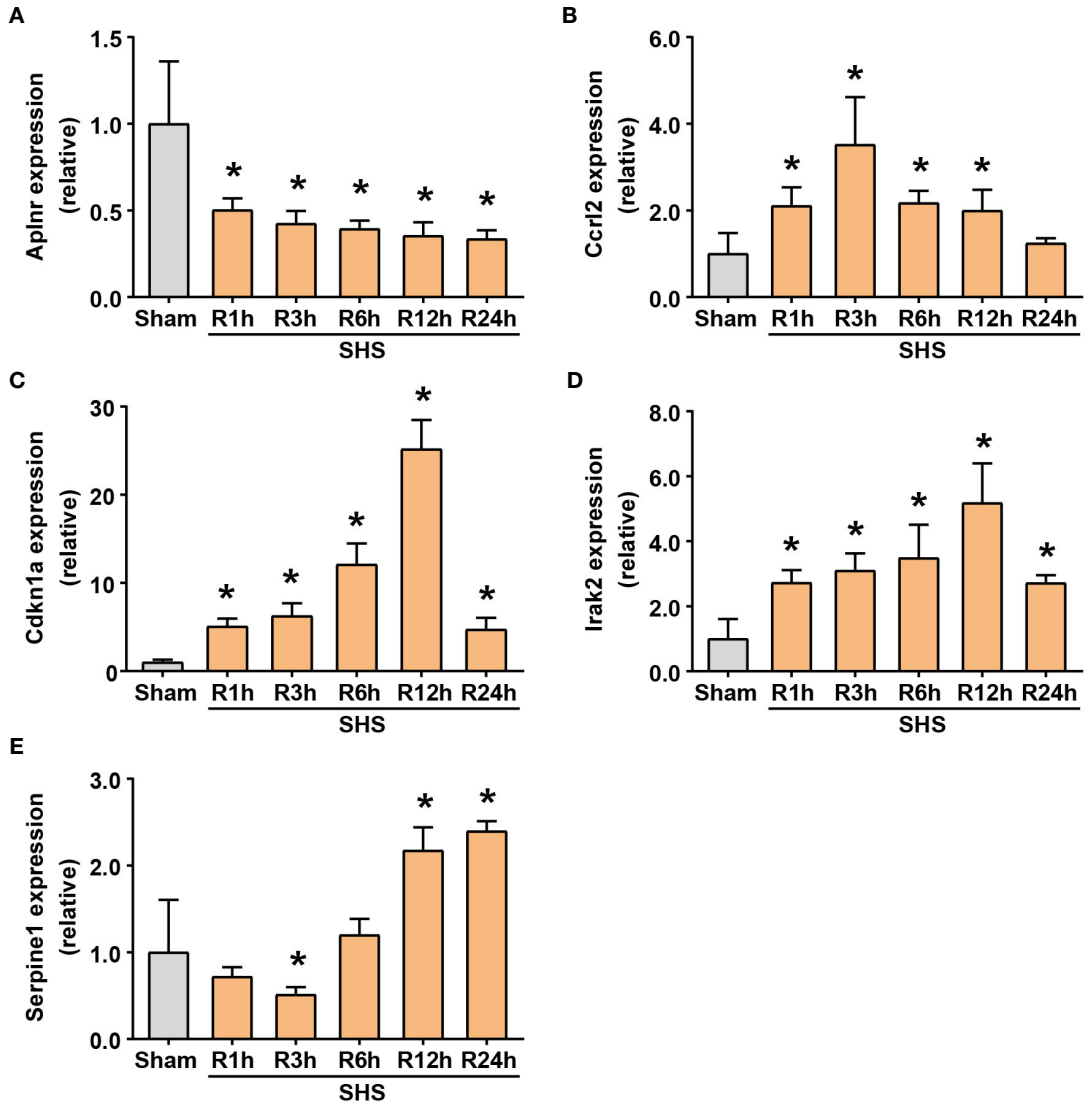
RT-qPCR of differentially expressing inflammation-related genes (DEIRGs) in myocardium of MCAO- and sham-treated mice. **(A–E)** Transcriptional expression of *Aplnr* **(A)**, *Ccrl2* **(B)**, *Cdkn1a* **(C)**, *Irak2* **(D)** and *Serpine1* **(E)** at 1, 3, 6, 12, and 24 h of perfusion or sham surgery. *P<0.05 compared with sham-treated mice.

Among them, *Aplnr* expression significantly decreased in MCAO-treated mice to 50% that of the sham group by 1 h after MCAO and continued to decrease thereafter. By 24 h, *Aplnr* transcription was approximately 30% of that detected in the sham mice (**Figure 7A**). By contrast, *Ccrl2* increased in the early stage of reperfusion, reaching >2-fold that of sham mice by 1 h post-MCAO, and peaking at 3.5-fold higher levels at 3 h, before decreasing to similar levels as sham controls by 24 h post-MCAO (**Figure 7B**). *Cdkn1a* and *Irak2* underwent similar increases, up to 5- and 2.7-fold of their expression compared to sham mice within 1 h after MCAO, respectively, and steadily increased until 12 h after MCAO. Notably, average *Cdkn1a* expression in the MCAO group reached approximately 25-fold its levels in comparison to the sham group, whereas *Irak2* transcript levels peaked at >5-fold that of the sham group. Although gradually decreasing by 24 h post-MCAO, transcript levels of *Cdkn1a* and *Irak2* were greater than 4.7 and 2.7 times, respectively, in MCAO mice compared to sham mice at the final time point (**Figures 7C, D**). However, *Serpine1* followed a different trend, decreasing to approximately 70% and 50% of its levels in comparison to sham mice at 1 h and 3 h, respectively. *Serpine1* transcription then increased, reaching approximately 1.2-, 2.2- and 2.4-fold that of its expression in the sham group at 6 h, 12 h, and 24 h after MCAO, respectively (**Figure 7E**).

These results verified that the expression of these inflammation-related genes indeed fluctuates significantly after MCAO, suggesting that MCAO-induced ischemic stroke may share a close relationship with cardiac injury. Although *Ccrl2* expression in MCAO mice did not significantly differ from its expression in comparison to the sham group at 24 h, the other four genes all had significantly different transcription levels from that of the sham group, leading us to classify them as target genes.

### 3.8 Western blot detection of target gene protein expression

**Figure 8:**
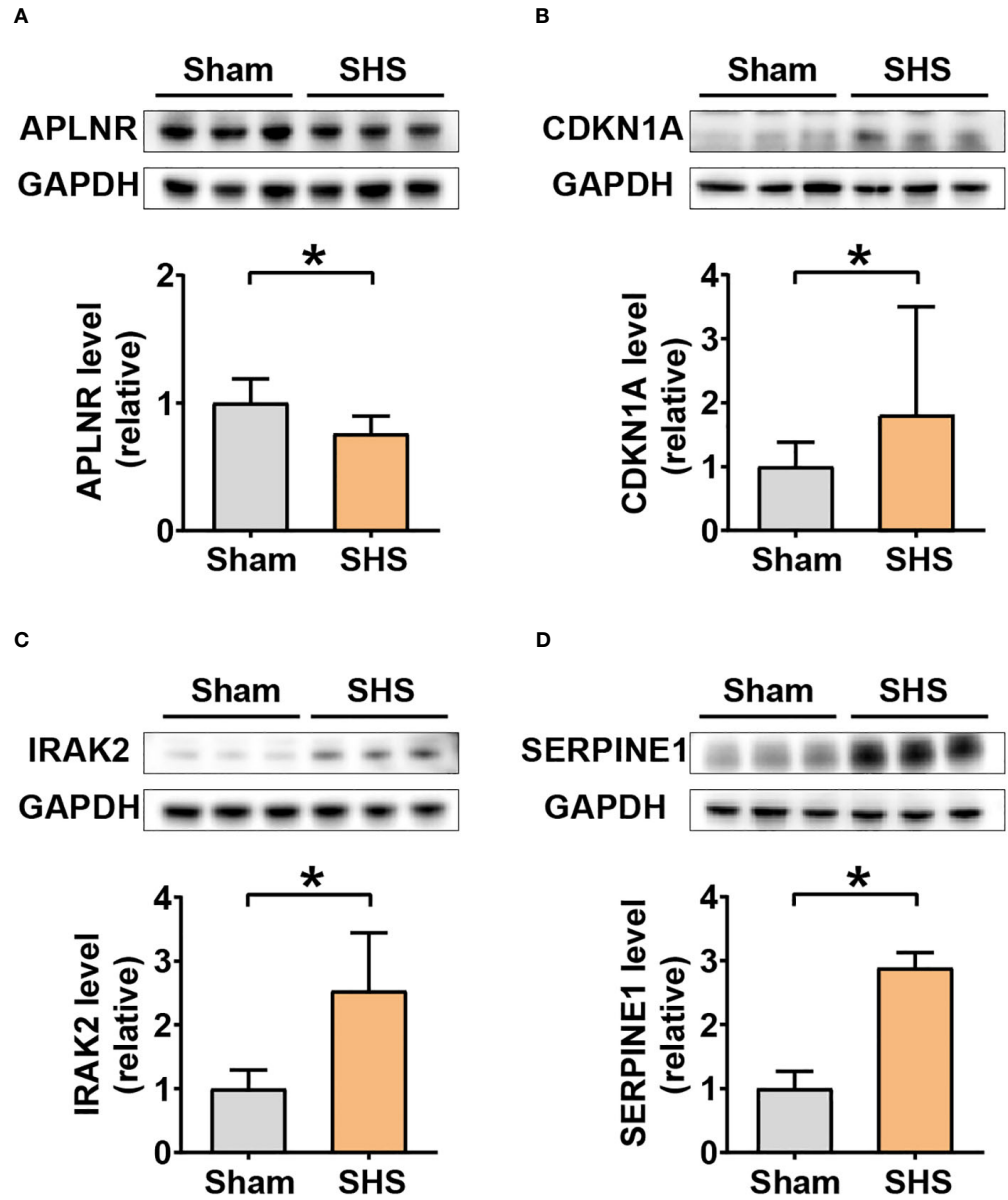
Western blot analysis of DEIRG protein levels in myocardium of MCAO- and sham-treated mice. Western blot image of bands (three replicates each) and quantitation of signal intensities normalized with GAPDH in fold-change for APLNR **(A)**, CDKN1A **(B)**, IRAK2 **(C)**, and SERPINE1 **(D)**. *P<0.05.

To further determine whether *Aplnr, Cdkn1a*, *Irak2*, and *Serpine1* could serve as candidate target genes, we examined their protein levels in heart tissue at 24 h post-reperfusion in MCAO- and sham-treated mice. The results showed that APLNR protein levels were significantly lower, while CDKN1A, IRAK2 and SERPINE1 accumulation was significantly higher in MCAO-treated heart tissue compared to that of sham controls (**Figure 8**).

## 4 Discussion

Accumulating clinical and experimental evidence suggests that cardiovascular complications are a major factor contributing to death after stroke. Unfortunately, the brain–heart cross-talk in response to ischemic stroke remains poorly understood, and a better understanding of clinical indicators of post-stroke cardiovascular complications is needed to improve clinical management and reduce mortality (1).

Veltkamp et al. (18) showed that inducing MCAO in mice for 60 min resulted in an early (24 h) and significant reduction in cardiac function with reduced left ventricular contractility and heart weight, accompanied by release of troponin and cardiomyocyte atrophy, confirming that cardiac injury can occur during the early stages of MCAO. Bieber et al. (7) performed a 30-minute transient occlusion of the middle cerebral artery in mice to induce cardiac dysfunction and initiated an 8-week course of treatment with metoprolol immediately after surgery. They found that early treatment of stroke could reduce heart damage and improve survival rate, prompting us to evaluate the gene expression profile of heart tissue early after MCAO to identify therapeutic targets for early intervention after ischemic stroke.

*Aplnr* encodes apelin receptors and is widely expressed in the brain, blood vessel, heart, cardiovascular system, and the central nervous system. APLNR regulates the normal physiological processes of these tissues, including cardiovascular response, angiogenesis, and the neuro endocrine response (19–21). APLNR signaling is associated with several pathologies, including ischemic stroke, cardiopathy, obesity, diabetes, and cancer (22), suggesting that APLNR function may have a crucial role in multiple physiological systems. In addition, the apelinergic system, comprising apelin and APLNR, is important in recovery after stroke by inhibiting neuronal apoptosis and promoting angiogenesis through various molecular pathways (23–25). These findings suggest that the APLNR may be a potential target for new treatments. The apelinergic system exhibits time imbalance during ischemic stroke (22), exclusively increasing in the ischemic phase, but decreasing in the reperfusion stage, which aligns well with results obtained in the current study. Agonists of apelin/APLNR have been previously shown to decrease infarct size in the heart only if specifically delivered during the reperfusion stage (26). Thus, a comprehensive, mechanistic understanding of the role of APLNR and its dynamic patterns of expression during ischemic stroke are necessary before testing potential therapeutic applications of APLNR agonists in a clinical setting.

The 7 transmembrane domain receptor protein, CCRL2, is primarily expressed on monocytes, macrophages, and dendritic cells, among other immune cell types, and is responsible for essential regulatory functions in immune signaling and inflammatory response, as well as infiltration and tumorigenesis. More specifically, CCRL2 is upregulated on activated dendritic cells and immune cells under pathological conditions to mediate inflammatory cell infiltration and other inflammation-related effects, but is suppressed in these immune cells in the resting state (27, 28). Previous studies have shown that CCRL2 activates T lymphocyte migration from the circulatory system to lymphatic tissue and sites of inflammation during immune response. This receptors also direct white blood cell infiltration and subsequent aggregation during inflammation, such as at infection sites, in a chemokine-dependent manner. In CCRL2-mediated immune response, CCRL2 receptors are activated upon recognition of infection or other pathogen- or wound-associated stimuli, and in turn, promote immune response to bolster host resistance mechanisms.

In our current study, early upregulation of *Ccrl2* transcription was observed in cardiac tissue following MCAO. However, its expression was restored to baseline (i.e., control group) levels by 24 h post ischemic stroke in mice. Therefore, we conclude that the *Ccrl2* gene may participate in the initial rapid inflammatory response to heart injury of MCAO mice, but its relatively brief activation suggests that it is may not be served as a reliable therapeutic target.

CDKN1A (cyclin dependent kinase inhibitor 1a), also known as p21, was the first-discovered member of the CKI protein family, and it is a relatively well-studied cyclin dependent kinase (CDK) inhibitor (29). In addition to participating in cell cycle regulation, CDKN1A also plays a regulatory role in cell aging, apoptosis, and different immune responses (30, 31). CDKN1A not only regulates the acquired immune response of T cells, but it also regulates innate immunity by modulating the activity of macrophages (32, 33). The mechanism of CDKN1A is complex and varies in different cell-type and stimulatory contexts. The function of CDKN1A can also be affected by the amount of DNA damage in a cell. When there is a low amount of DNA damage, CDKN1A expression increases to block the cell division cycle and prevent apoptosis. By contrast, when there is a high level of DNA damage, CDKN1A expression decreases, inducing apoptosis (34). CDKN1A is highly expressed in myocardial cells and has been found to play an important role in inflammatory diseases of the heart (35). Clarifying the changes in expression of CDKN1A during SHS will help verify its role in SHS pathogenesis to potentially uncover novel treatment strategies. QPCR-based relative expression analysis in the current study showed that *Cdkn1a* expression underwent the most dramatic changes following MCAO, increasing to 25-fold that detected in the sham group after 12 hours of reperfusion. Furthermore, although *Cdkn1a* expression decreased after this time point, it showed sustained upregulation, maintaining 4.7-fold higher transcription than that in sham controls at 24 hours after MCAO. These findings suggest that *Cdkn1a* could potentially serve as an effective target for SHS treatment.

IRAK2 acts upstream of or within the lipopolysaccharide-mediated signaling pathway and helps to regulate immunity and inflammation (36). IRAK2 is responsible for the activating signal from immune cells mediated by adaptor molecules TRIF and MyD88. IRAK2 mediates nuclear export of inflammatory genes as well as regulates downstream signaling pathways to promote expression of inflammatory genes (37, 38). Liu et al. (39) found that circulating interleukin-1β promotes endoplasmic reticulum stress-induced apoptosis of myocytes in diabetic cardiomyopathy via IRAK2. In our recent ongoing research, we found that interleukin-1β mRNA expression appears to be upregulated in cardiac tissue after MCAO (unpublished data), which is consistent with findings reported by Yan and co-workers (40). These data suggest that the accumulation of interleukin-1β in cardiac tissue after SHS may be associated with increased *Irak2* levels and might contribute to the pathological development of SHS. These studies collectively demonstrate that IRAK2 is involved in ischemic reperfusion following MCAO and that inhibiting IRAK2 may potentially confer therapeutic benefits.

SERPINE1 is secreted into plasma in an active state and contributes to processes such as fibrinolysis, inflammatory response, complement activation, and coagulation in human connective tissue (41–44). SERPINE1 also protects the cell membrane from enzymatic degradation by plasma, thus maintaining structural integrity, and may also affect nerve growth, collagen activation, and vascular repair (45). These previously characterized functions may explain, at least in part, why we observed a rapid decrease in *Serpine1* expression after MCAO in mice. Other studies have shown that increased SERPINE1 levels are closely related to insulin resistance, obesity, cardiovascular and cerebrovascular diseases, type 2 diabetes, hypertension, and lipid metabolism disorders (42, 43), which could also partially explain the observed increase in SERPINE1 at the mRNA and protein levels at 24h post-MCAO. Thus, modulation of SERPINE1 levels may be a promising strategy for stimulating angiogenesis, reducing blood–brain barrier dysfunction, and treating cardiovascular injury (45, 46).

We verified the MCAO-specific changes in expression of four genes involved in the inflammatory response using bioinformatics analysis and *in vivo* experiments, but our study has some limitations. First, the number of samples in the public RNA-seq dataset was relatively small, and additional sequencing data are needed to validate our findings. Second, due to mild inflammation in the myocardium of sham-treated animals, some statistical data are missing, and a large number of samples is needed for a robust analysis of the immune infiltrate in the myocardium of mice after MCAO. Third, flow cytometry are needed to purify the populations and validate the gene expression changes. We will strive to address these limitations in our future research.

In conclusion, we investigated the impact of ischemic stroke on cardiac changes and evaluated changes in gene expression profiles in the heart caused by stroke damage. An improved understanding the inflammatory molecules, signaling pathways, and immune cells involved in the pathological process of SHS may uncover new treatment strategies that could improve clinical outcomes of patients after ischemic stroke.

## Data availability statement

The datasets presented in this study can be found in online repositories. The names of the repository/repositories and accession number(s) can be found in the article/supplementary material.

## Ethics statement

The animal study was approved by Animal Ethical and Welfare Committee. The study was conducted in accordance with the local legislation and institutional requirements.

## Author contributions

JZ performed the bioinformatic analysis, analyzed the data and wrote the manuscript. YM made the MCAO model and performed the *in vivo* experiment. XG and JW conceived and designed the experiments, revised manuscript and offer funding supports.
